# Changes in the Mitochondria in the Aging Process—Can α-Tocopherol Affect Them?

**DOI:** 10.3390/ijms241512453

**Published:** 2023-08-05

**Authors:** Gaetana Napolitano, Gianluca Fasciolo, Maria Teresa Muscari Tomajoli, Paola Venditti

**Affiliations:** 1 Department of Science and Technology, University of Naples Parthenope, Via Acton n. 38, I-80133 Naples, Italy; gaetana.napolitano@uniparthenope.it (G.N.); mariateresa.muscaritomajoli001@studenti.uniparthenope.it (M.T.M.T.); 2 Department of Biology, University of Naples ‘Napoli Federico II’, Complesso Universitario di Monte Sant’Angelo, Via Cinthia, I-80126 Naples, Italy; gianluca.fasciolo@unina.it

**Keywords:** ROS production, oxidative damage, mitochondrial dynamics, mtDNA, inflamm-aging, vitamin E, vitamin E metabolites

## Abstract

Aerobic organisms use molecular oxygen in several reactions, including those in which the oxidation of substrate molecules is coupled to oxygen reduction to produce large amounts of metabolic energy. The utilization of oxygen is associated with the production of ROS, which can damage biological macromolecules but also act as signaling molecules, regulating numerous cellular processes. Mitochondria are the cellular sites where most of the metabolic energy is produced and perform numerous physiological functions by acting as regulatory hubs of cellular metabolism. They retain the remnants of their bacterial ancestors, including an independent genome that encodes part of their protein equipment; they have an accurate quality control system; and control of cellular functions also depends on communication with the nucleus. During aging, mitochondria can undergo dysfunctions, some of which are mediated by ROS. In this review, after a description of how aging affects the mitochondrial quality and quality control system and the involvement of mitochondria in inflammation, we report information on how vitamin E, the main fat-soluble antioxidant, can protect mitochondria from age-related changes. The information in this regard is scarce and limited to some tissues and some aspects of mitochondrial alterations in aging. Improving knowledge of the effects of vitamin E on aging is essential to defining an optimal strategy for healthy aging.

## 1. Introduction: An Overview of Aging Theories and the Mitochondrial Theory of Aging

Aging is defined as a progressive time-dependent cellular functional decline that increases susceptibility to different types of stress and is associated with an increased risk of many diseases, including cancer, diabetes, and cardiovascular and neurodegenerative disorders [1].

Some theories explaining the progression of aging exclude the possibility that such a process could be studied scientifically or manipulated [2]. Indeed, the evolutionary theory considers aging to be caused by the concomitant disruption of multiple biological systems due to the diminishing influence of post-reproduction natural selection [2]. This theory was based on the observation that since most animals in the wild do not reach old age, there has been little pressure during evolution to select alleles that confer many benefits later in life [3]. Thus, the many harmful phenotypes we commonly associate with aging have been attributed to the diminished influence of natural selection. Evolutionary theory has also postulated that aging arises because of antagonistic pleiotropy, in which alleles that provide substantial benefits early in life become harmful during adulthood [4]. In summary, evolutionary theory views aging as an inevitable by-product of the rules of natural selection and not as a regulated process amenable to scientific study. Evolutionary theory was challenged when it emerged that the shape of the mortality curves for widely divergent organisms, ranging from unicellular yeast to humans, is similar in almost all organisms, showing higher mortality rates with advanced age [5]. This observation suggested a commonality in the aging process that is potentially open to scientific study, particularly in model organisms. Furthermore, it has been found that, unlike aging itself, the aging rate can be regulated. Thus, closely related species show very different life spans, i.e., in the rodent family, where a typical rat lives about five years and naked mole rats live about thirty years. Furthermore, even within the same species, significantly different rates of aging have been observed. A clear example of this is found in honeybees, where the queen and worker bees are genetically identical, but the average life span of the queen is ten times longer than that of the workers, suggesting the importance of epigenetics in the aging process [2].

Nowadays, it has been established that aging is a complex trait controlled by genes and the environment. López-Otín proposed nine hallmarks of aging: genomic instability, telomere attrition, epigenetic alterations, loss of proteostasis, deregulated nutrient sensing, mitochondrial dysfunction, cellular senescence, stem cell exhaustion, and altered intercellular communication [6]. These hallmarks are generally thought to contribute to the aging process and, together, determine the aging phenotype. Every hallmark should ideally meet certain criteria: (1) it should manifest itself during normal aging; (2) experimental aggravation is expected to accelerate aging; and (3) its experimental improvement should delay the normal aging process and thus increase a healthy lifespan [6]. These requirements are met to varying degrees by the proposed hallmarks, but the last criterion is the most difficult to achieve, even though the broad interconnectedness of the hallmarks implies that experimental improvement of one hallmark may affect the others.

It has been found that the target of the rapamycin signaling pathway (mTOR) plays a crucial role in the regulation of some of the hallmarks of aging [6]. mTOR is a major regulator of lifespan in all eukaryotes and is also considered to be the mediator of some of the effects of dietary restriction, the only intervention that reduces age-related causes of mortality [7] and can determine a prolongation of lifespan [8]. In many diseases related to aging, such as cancer, neurodegeneration, and auto-immunity, deregulation of the mTOR signaling pathway is found [9]. It has been suggested that mTOR acts as a rheostat of energy, able to detect different inputs such as amino acids, oxygen, hormones, and stress, and regulate lifespan by regulating several functions in the cell such as gene expression, ribosome biogenesis, proteostasis, and mitochondrial metabolism. Genetic or pharmacological actions that decrease the activity of mTOR result in the upregulation of stress responses, changes in mitochondrial oxygen consumption and metabolism, and decelerate the rate of aging, prolonging the lifespan.

Therefore, mitochondria seem to play an important role in aging and are critical for cellular function and homeostasis. They are involved in various cellular processes, including the fine control of calcium balance, the metabolism of energy substrates during feeding and fasting, and cell signaling pathways under stressful conditions. Moreover, mitochondria show a heterogeneous structure due to the balance between the two opposite processes of fusion and fission of the mitochondrial membranes [10]. Microscopic observation of live cells revealed that mitochondria are organized in a cellularly interconnected network and are dynamic [10]. They are continuously moving along the cytoskeleton and frequently fuse and divide, which controls their morphology and distribution in the cell. The loss of fusion or fission activities generates dysfunctional mitochondria, suggesting that mitochondrial shape and function are intimately linked [11]. 

The efficiency of mitochondria progressively decreases with age, and they become potentially toxic to cells [12]. Although the relationship between aging and mitochondrial dysfunction has long been studied, the mechanisms underlying this relationship have not been fully defined, which represents a challenge for aging research.

One of the most popular theories that link mitochondria and cellular dysfunctions in aging is the “mitochondrial free radical theory of aging” (MFRTA) [13], which has its roots in Pearl’s “rate of living” theory [14]. Pearl suggested that the primary determinant of how long species live is influenced by the relative speed of their resting metabolism. That is, metabolic rate is thought to be inversely proportional to maximum life span, meaning that species that live fast will die young while those that have a slower metabolic rate will live slower and longer. The mechanism underlying such a relationship was identified in the production of reactive oxygen species (ROS) as by-products of cellular metabolism [15]. ROS can damage lipids, proteins, DNA, and, therefore, cellular components, determining loss of cellular function. When it was suggested that mitochondria are the main intracellular sites of ROS production, the theory was defined as the “mitochondrial free radical theory of aging” [13]. Such a theory suggests that the persistent mitochondrial ROS production during life causes oxidative damage to proteins, lipids, and DNA, which can accumulate, contributing to aging and age-related diseases in an inevitable but stochastic process. 

Since cellular aging seems to be caused by the molecular alterations induced by ROS and the consequent dysfunctions, it has been hypothesized that antioxidant substances capable of effectively extinguishing various forms of ROS or of reducing their production, especially in the mitochondria, can slow down the speed of aging and reduce the incidence of age-related diseases [16]. On this assumption, numerous studies have been conducted aimed at estimating the effects of levels of endogenous antioxidants or the effect of the administration of exogenous antioxidants on aging and on age-related processes and diseases [17,18]. Until now, the outcomes of the effects of the administration of single or multiple antioxidants on the age-related decline of brain functions have been inconclusive, and other studies are necessary to define the efficacy of antioxidant supplementation [17]. 

Some experimental evidence, mainly obtained in model animals for aging studies, suggests that antioxidant supplementation can extend lifespan. For example, in Drosophila, a widely used model for biological research, both melatonin and resveratrol extend lifespan [19,20,21]. The effects of antioxidants on the extension of lifespan have also been evaluated in other animal models, furnishing divergent results [22,23]. Moreover, the effects seem to depend on the dose of antioxidants tested. For example, Drosophila fed a diet supplemented with 1 mg/mL of N-acetylcysteine showed an increased lifespan, while those supplemented with 10 mg/mL of N-acetylcysteine showed a reduced lifespan [24].

Furthermore, the relationship between decreased ROS levels and longevity is complicated by the observation that treatment with compounds that increase mitochondrial ROS production, such as 2-deoxy-d-glucose [25] or complex I inhibitors [26], extends lifespan in Caenorhabditis elegans. Similar results were obtained in mice treated with d-glucosamine, an inhibitor of glycolysis that induces an increase in mitochondrial ROS production. The increased life span in d-glucosamine-treated mice was prevented by antioxidant administration [27]. 

ROS can exert beneficial or detrimental effects on longevity, depending on the species and conditions [28]. The beneficial effects of ROS can be explained in the context of the mitochondrial hormesis (mithormesis) theory [29]. Indeed, while the Free Radical Theory of Aging assumes the existence of a linear dose-response relationship between increasing amounts of ROS, oxidative stress, and the incidence of mortality, mithormesis theory assumes a non-linear dose-response relationship, with decreased incidence of mortality at low ROS levels and increased incidence of mortality at higher ROS levels [29]. In other words, low levels of ROS can induce an adaptive response to promote an endogenous defense mechanism, alleviating the susceptibility to increased ROS levels.

A more recent vision of the mitochondrial role in the cells is that, besides being the sites of production of metabolic energy, they are also regulatory hubs able to coordinate vital physiological processes both at the level of the cell and the whole organism. Information concerning proteotoxic and metabolic stress and inflammatory signals is sent to the nucleus from mitochondria to maintain cellular homeostasis [30]. Several molecular compounds are involved in mitochondrial communication, including proteostasis signaling molecules, mitochondrial metabolites, and mitokines such as mitochondrial-derived peptides (MDPs). Multiple age-related mitochondrial dysfunctions affect communication processes, induce maladaptive metabolic shifts, and reduce organismal fitness. Thus, they contribute to aging phenotypes and age-related disabilities [30].

Therefore, an excess of antioxidants can have a negative effect, presumably by preventing the hormetic response. Antioxidant supplementation with vitamin C, vitamin E, or a combination of both antioxidants can impair the whole-cell adaptive response to changes in the environment, impairing the activation of factors involved in such adaptations [31,32,33,34]. 

In this review, we will furnish an overview of mitochondrial involvement in the aging process and the capacity of antioxidant molecules, particularly vitamin E, to prevent age-linked mitochondrial dysfunctions.

## 2. Mitochondrial Production of ROS and Their Role in Aging

The main role of the mitochondria in the cell is to produce metabolic energy as adenosine triphosphate (ATP) through the process of oxidative phosphorylation (OXPHOS). OXPHOS occurs in the inner mitochondrial membrane, which contains the membrane electron transfer chain (ETC). 

The catabolism of lipids, proteins, and carbohydrates leads to the formation of the acetyl groups of the acetyl coenzyme A that is oxidized in Krebs’s cycle to CO_2_ with the formation of the reduced form of the nicotinamide adenine dinucleotide (NADH) and flavin adenine dinucleotide (FADH2). NADH and FADH2 pass the electrons to the ETC components and eventually to oxygen. The electron transfer is a process that occurs in several stages that gradually release the energy linked to the drop in the electron potential. ETC consists of four protein complexes that transfer electrons, acting as enzymes that carry out oxidation and reduction reactions, allowing the transfer of electrons from one complex to the next in series. The complexes are named: Complex I, II, III, and IV, or NADH dehydrogenase, succinate dehydrogenase, ubiquinol-cytochrome c reductase, and cytochrome c oxidase, respectively. Complex V, F_1_F_0_-ATP synthase, is functionally different, facilitating the synthesis of ATP rather than electron transfer. Complex I, III, and IV pump protons into the intermembrane space. The mechanism of proton pumping involves conformational changes in the protein complexes, whose exact mechanism is still under research [35].

Complex I and Complex II receive electrons from NADH and FADH2, respectively, and transfer them to ubiquinone (coenzyme Q, CoQ), which transfers electrons through Complex III to cytochrome c (Cytc) [36].

Complex IV removes electrons from four Fe^2+^ heme molecules of reduced Cytc molecules, which are oxidized to Fe^3+^-Cytc, transferring them to oxygen, the final electron acceptor. In this way, oxygen forms water by tetravalent reduction.

$$O_{2}+e^{-}\to O_{2}^{•-}+e^{-}+2H^{+}\to H_{2}O_{2}+e^{-}+1H^{+}\to H_{2}O+{}^{•}OH+e^{-}+1H^{+}\to H_{2}O$$

It has been estimated that approximately 90% of mammalian oxygen consumption in standard conditions (i.e., measured on an adult individual, awake but resting, stress-free, not digesting food) is mitochondrial, of which approximately 20% is uncoupled due to the mitochondrial proton leak and 80% is coupled to ATP synthesis [37].

The transfer of electrons along the inner mitochondrial ETC also produces ROS [35]. ROS include highly reactive molecules, such as the hydroxyl radical (^•^OH), and molecules that are less reactive, such as superoxide (O_2_^•−^) and hydrogen peroxide (H_2_O_2_), that can oxidize cellular constituents, damaging them and affecting their functionality [35]. 

During the tetravalent oxygen reduction by Complex IV of the ETC, ROS release in the surrounding medium is avoided due to the structure of the cytochrome oxidase, which facilitates a complete reduction of O_2_ [38]. Conversely, other carriers of the ETC can generate, by auto-oxidation and univalent O_2_ reduction, the superoxide that is released into the matrix or the inter-membrane space of the mitochondria. It is long known that such autoxidizable carriers are present in both Complex I [39,40] and Complex III [41]. 

In Complex I, the quinone binding site is also responsible for producing most of the superoxide or hydrogen peroxide in the presence of succinate or glycerol 3-phosphate as respiratory substrates. In these conditions, the generation of superoxide is due to the reverse transport of electrons (ROS-RET), which depends on the high ubiquinol/ubiquinone ratio and the high protonmotive force that pushes electrons into Complex I against the redox potential determining the formation of NADH from NAD^+^ [42]. It has been found that increased ROS and decreased NAD^+^ levels are frequently observed in aging and age-related diseases and that inhibition of RET can protect against age-linked alterations [43].

ROS production sites have also been identified in Complex II [44,45] and in several mitochondrial enzymes [46,47,48]. Currently, the in vivo sites of ROS generation are an unsolved problem.

The metabolic state of the mitochondria can influence their ROS production. Under conditions where the ATP/ADP ratio is high, the flow of electrons along the mitochondrial complexes is low, but the proton gradient, mitochondrial inner membrane potential (MMP), and the degree of reduction of autoxidizable carriers are high. In such conditions, the loss of electrons can be more significant, favoring ROS production. It should be emphasized that electron leakage is not directly dependent on mitochondrial membrane potential or proton motive force unless the altered proton motive force affects electron transport and alters the concentration of the autoxidizable electron carriers in the reduced state [48]. Uncouplers and ADP, which stimulate ATP synthesis, increase the respiration rate, lower membrane potential, and decrease the production of ROS in isolated mitochondria [49]. Uncoupling proteins (UCPs) are inner membrane carrier proteins that can induce proton leakage and dissipate proton motive force. Several pieces of evidence suggest that mild uncoupling is mediated by uncoupling proteins 2 and 3 (UCP2 and UCP3) that control the production of ROS, decreasing membrane potential [49], therefore reducing oxidative damage [50].

Superoxide (O_2_^−^) is converted into hydrogen peroxide (H_2_O_2_) spontaneously or by a reaction catalyzed by superoxide dismutase (SOD) enzymes located in the mitochondrial matrix (SOD2, or MnSOD) and the cytoplasm (SOD1, or Cu-ZnSOD) [51]. H_2_O_2_ can give rise to the highly reactive hydroxyl radical (^•^OH) in the presence of ferrous iron by the Fenton reaction [52]. 

Mitochondrial ROS also act as fundamental cellular messengers in controlling cellular homeostasis [30,53]. It has been reported that the suppression of ROS-RET production under stress conditions shortens survival in both fruit flies [54] and mice [55], preventing an adaptative transcriptional response. Conversely, stimulation of ROS-RET production in basal conditions extends the lifespan of Drosophila [56]. These results suggest that a mitochondrial redox signaling pathway exists that acts to regulate lifespan under both basal and stress conditions. It has recently been shown that the mitochondria of young Drosophila increase the production of ROS-RET in response to specific stimuli such as heat stress, while the mitochondria of old Drosophila, due to the reduced flow of electrons, continuously produce high levels of ROS, which reduce the redox signaling capacity [57].

While low ROS production is important for target-specific redox signaling, high ROS production leads to reduced redox signaling and/or damage to biomolecules [58]. Oxidative stress originates from the imbalance between the production of ROS and the ability of the antioxidant system to counteract their oxidative action; in this case, the redox signaling is altered and increases oxidative damage to macromolecules [59]. Indeed, ROS can damage lipids, DNA, and proteins [60].

Mono- and polyunsaturated lipids are the molecules more prone to oxidation, which results in the formation of lipid hydroperoxides (LOOH) as primary nonradical reaction products. LOOH may undergo degradation into various products that have been implicated in vital biological reactions and in the pathogenesis of various diseases [61]. The molecular changes caused by the reactions of products of lipid peroxidation alter the structure and function of proteins and DNA and are responsible for the cytotoxicity induced by these molecules [61].

ROS can oxidize DNA, modifying the structure of nucleotides and inducing double-stranded break formation, which can favor genomic instability [62]. Moreover, ROS can directly oxidize nucleoside bases (e.g., formation of 8-oxo-7,8-dihydro-2′-deoxyguanosine), which can determine G-T or G-A transversions if unrepaired [63]. The base excision repair pathway recognizes, and repairs oxidized bases, but when oxidation occurs simultaneously on opposing strands, the base excision repair system can determine the generation of a DNA double-strand break, which can be mutagenic due to chromosomal rearrangements or loss of genetic information [64]. ROS accumulation also induces mitochondrial DNA (mtDNA) lesions, strand breaks, and degradation [65].

ROS can directly or indirectly affect protein functionality. The direct action of ROS determines the nitrosylation, carbonylation, disulfide bond formation, and glutathionylation of proteins [66]. Indirectly, oxidative damage to proteins results from their conjugation with products of the breakdown of oxidized fatty acids [66]. Oxidatively damaged proteins undergo site-specific modifications of amino acids, fragmentation of the peptide chain, aggregation of cross-linked reaction products, altered electric charge, and increased susceptibility to proteolysis [66]. 

Since the content of oxidized macromolecules shows an age-dependent increase, these are considered biomarkers of the aging process [67]. This idea agrees with the MFRTA, which explains aging due to the accumulation of damage to macromolecules. The damage to the macromolecules is due to ROS produced during the metabolic process essential for cells that occurs in the mitochondria [15], and the aging process is inherently determined as ROS production is continuous as they are by-products of the essential process of ATP synthesis. Therefore, aging is essentially due to the mitochondrial production of ROS, thus underlying the relationship between lifespan and rate of oxygen consumption through the mitochondrial respiratory chain, which is the major source of ROS.

So far, it has been found that two factors strictly correlate with animal longevity in vertebrates, including mammals and birds, namely the rate of mitochondrial ROS production [64] and the degree of unsaturation of the fatty acids at the cellular and mitochondrial level [68]; the longer the longevity of a species is, the smaller these two parameters are. In long-lived animal species, decreased mitochondrial ROS production decreases mitochondrial oxidative damage [68]. The lower content of double bonds in fatty acids decreases the susceptibility to free radical damage, decreasing the peroxidability index [68].

At the level of a single organism, aging goes hand in hand with an increase in ROS production [69] and oxidative damage to macromolecules [70]. Moreover, oxidative stress has been found in several age-related diseases [60]. A common feature of the markers of aging is the alteration of mitochondrial functionality that is involved in increased oxidative stress, which is associated with the aging process [71,72,73]. 

## 3. mtDNA and Aging 

One of the most relevant features of mitochondria is that they possess their own DNA (referred to as mtDNA) that codes for some of the proteins that are components of the ETC (13 in humans and 8 in yeast) [74]. The genetic information for the four complexes and free electron transporters is contained in both mtDNA and nuclear DNA [74].

Unlike nuclear chromatin, mtDNA is not associated with histones in the form of nucleosomes but is packaged into nucleoids, structures composed of mtDNA and numerous nucleoid-associated proteins [75]. Nucleoids are roughly spherical, approximately 100 nm in diameter, and contain several copies of mtDNA [75]. They are close to the ETC, and increased ROS generation can affect mtDNA base modifications, including the formation of abasic sites, single- and double-strand breaks, point mutations, and large deletions [76].

Several pieces of evidence suggest that an accumulation of mtDNA mutations during aging [77,78] occurs, even if it has been pointed out that oxidative damage in both model organisms and human tissues is not as high as expected [79,80,81]. Therefore, the debate as to whether the accumulation of damaged mtDNA is random or related to the aging process is still ongoing. Furthermore, the accumulation of mtDNA mutations in somatic cells seems to be due to errors during replication attributable to dysfunctions in mitochondrial DNA polymerase γ. This idea is supported by the observation that expression of a mutant DNA polymerase γ in mice induces proofreading defects during mtDNA replication, increases the burden of mtDNA mutations in both homozygous and heterozygous mice, and is associated with premature aging [79,82]. Experiments performed in *C. elegans* suggested that mtDNA mutation alone does not determine lifespan [83]. Indeed, only mice homozygous for the DNA polymerase γ mutation, which exhibit a very high mtDNA mutation burden, have a shortened life span [84,85]. High mtDNA mutations in mice have been shown to be associated with slow nuclear DNA replication fork progression, cell cycle stalling, and chronic stress in DNA replication, leading to double-stranded DNA breaks in proliferating progenitor or stem cells [86]. The mechanism underlying these effects involves increased mtDNA replication frequency, sequestration of nucleotides in mitochondria, depletion of total cellular nucleotide pools, decreased availability of deoxynucleoside 5′-triphosphate (dNTP) for nuclear genome replication, and impairment of nuclear genome maintenance [86]. These data suggest that defects in mtDNA replication may challenge the stability of the nuclear genome and that mtDNA mutations contribute to organismal aging [86].

## 4. Mitochondrial Turnover and Aging

### 4.1. Mitochondrial Fusion and Fission

Mitochondria are subject to continuous rearrangement and turnover that aim to preserve their functionality. Maintaining a healthy mitochondrial population is crucial to safeguarding cell function and the energy homeostasis of the whole body.

The rate at which the mitochondrial pool renews depends on the balance between mitochondrial biogenesis and fusion, fission, or degradation through mitophagy processes. The processes of biogenesis and mitophagy generate new mitochondria and eliminate them, respectively. Mitophagy is relevant both for the adaptation of the mitochondria’s content to the cell’s metabolic needs and the removal of damaged organelles. Furthermore, several data points suggest that mitochondrial fission followed by selective fusion is a prerequisite for the degradation of dysfunctional mitochondria via autophagy [87]. Alterations in the delicate balance of the processes involved in mitochondrial turnover (mitophagy, mitochondrial biogenesis, and mitochondrial dynamics) can play a crucial role in the aging process [88]. In nematodes, mitochondria gradually accumulate with age, and depletion of the master regulator of autophagy recapitulates the effects of aging on mitochondrial mass in young adult animals [89]. The impairment of mitophagy reduces the resistance to stress and activates a signaling pathway that, starting from the mitochondria, regulates genes involved in both mitochondrial biogenesis and mitophagy [89]. These data suggest the existence of a feedback loop capable of integrating metabolic signals to coordinate mitochondrial biogenesis and turnover. The uncoupling of these two processes during aging contributes to the excessive accumulation of damaged mitochondria and the decline of cellular function [89].

For the understanding of the molecular mechanisms underlying mitophagy, the discovery of the pathway mediated by phosphatase and tensin homolog (PTEN)-induced putative kinase 1 (PINK1)-Parkin was crucial [90,91,92]. The protein PINK1 was identified in 2001 [93]. It is a serine/threonine kinase that possesses a mitochondrial targeting sequence at the N-terminal site and a signal that allows localization at the outer mitochondrial membrane (OMM) [94]. Parkin was discovered in 1998 and is a ubiquitin E3 ligase whose name is linked to its relevant role in the pathogenesis of juvenile parkinsonism [95]. 

Briefly, PINK1 is continuously degraded in a complex process that includes its importation into the mitochondrial matrix, where it undergoes cleavage by both mitochondrial processing peptidase (MPP) and presenilin-associated rhomboid-like protease (PARL). Then, PINK1 is translocated back to the cytosol and degraded via the N-end rule pathway [96].

When mitochondrial damage occurs, such as a fall in membrane potential or accumulation of misfolded proteins, the PINK1- and Parkin-dependent mitophagy pathways are activated [91]. PINK1 is stabilized at the OMM, where it undergoes autophosphorylation and then phosphorylates Parkin, activating Parkin ligase E3Ub activity and its recruitment to damaged mitochondria [87]. Activated Parkin adds ubiquitins to several mitochondrial substrates, including mitofusin 1 and mitofusin 2 (MFN1/2) [97], voltage-gated anion channel 1 (VDAC1) [98], PARIS [99], and Miro [100]. Polyubiquitination of mitochondrial proteins induces association with autophagy receptors, thereby resulting in the formation of the autophagosome [101,102], which subsequently fuses with lysosomes, thus favoring the degradation of mitochondria [103].

In summary, damaged mitochondria are identified and labeled by parkin-dependent ubiquitination of certain proteins and eliminated. However, there is also an alternative pathway for mitophagy initiation since PINK1 can directly recruit autophagy receptors independently of Parkin [103]. 

**Figure 1:**
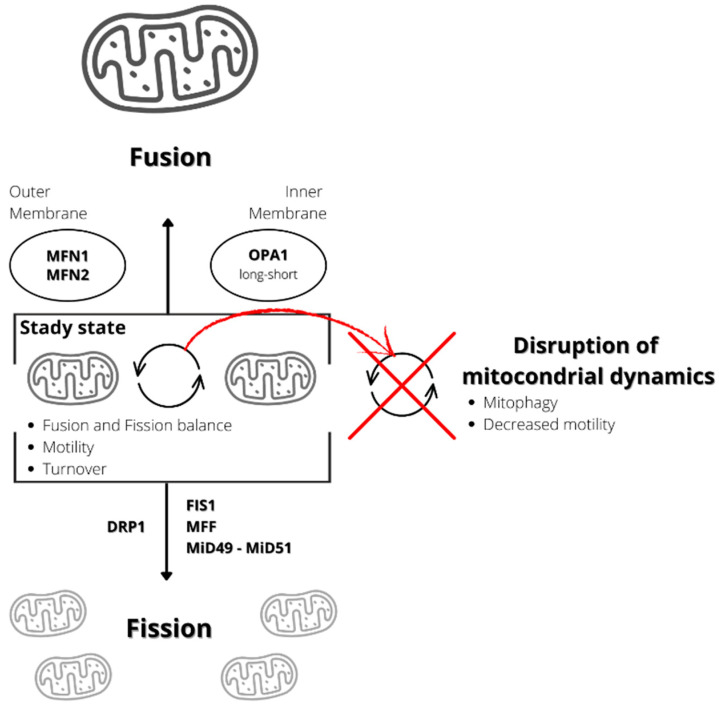
Schematization of mitochondrial dynamic. Mitochondrial fission produces two mitochondria from one mitochondrion, while fusion determines the production of one mitochondrion from two. Proteins involved in the fission processes are dynamin-related protein 1 (DRP1), fission 1 homolog protein (FIS1), mitochondrial fission factor (MFF), mitochondrial dynamics protein of 49 kDa (MiD49), and mitochondrial dynamics protein of 51 kDa/mitochondrial elongation factor 1 (MiD51/MIEF1). The proteins involved in the fusion process are mitofusins 1 and 2 (MFN1 and MFN2), involved in the outer membrane fusion, and OPA1 (long and short forms), involved in the intra-membrane fusion. Disruption in the fusion/fission balance can reduce mitochondrial motility, inducing mitophagy.

Moreover, mitophagy is tightly regulated by the processes of fusion and fission, also indicated as “mitochondrial dynamics” [104] (Figure 1). Fusion occurs when two adjoining mitochondria merge, forming a longer mitochondrion, while fission occurs when one mitochondrion separates into two [105].

Fusion and fission processes are both regulated by specific proteins. The proteins involved in the process of fusion are three GTPases, two of which localize at the outer mitochondrial membrane, MFN1 and MFN2, and one located at the inner mitochondrial membrane and intermembrane space, optic atrophy gene 1 (OPA1) [106]. Fusion determines the mixing of the content of mitochondrial matrixes, intermembrane spaces, and the mtDNA molecules of two mitochondria. This process can be relevant to buffering, at least partially, defects and transient stresses. Indeed, it enables optimal mitochondrial function by diluting mutated mtDNA and salvaging damaged mitochondria by acquiring key components from healthy mitochondria [107]. 

The proteins involved in mitochondrial fission include a soluble cytosolic protein, the dynamin-related protein 1 (DRP1), a member of the dynamin family of GTPases, and mitochondria-bound proteins, including fission 1 homolog protein (FIS1), mitochondrial fission factor (MFF), mitochondrial dynamics protein of 49 kDa (MiD49), and mitochondrial dynamics protein of 51 kDa/mitochondrial elongation factor 1 (MiD51/MIEF1) [106]. Mitochondrial outer membrane contact sites with the endoplasmic reticulum that define the position where the constriction machinery assembles, and division occurs regulate the process [108,109,110,111]. DRP1 is not anchored to the membrane and is recruited from the cytoplasm to homo-oligomerize around the outer mitochondrial membrane to constrict it in a process that GTP binding and hydrolysis drive [112]. 

Following a fission event, the daughter mitochondrion may either maintain intact membrane potential or depolarize. If it depolarizes, it is unlikely to proceed to a subsequent fusion unless it repolarizes. After being depolarized and solitary for a few hours, the mitochondrion is removed by autophagy [113].

Mitochondrial dynamics regulate mitophagy and contribute to mitochondrial quality control. In mouse pancreatic β-cells, mitochondrial fission results in the identification of damaged mitochondria in the mitochondrial population and their subsequent removal by mitophagy [113]. Thus, defects in mitochondrial fusion or fission processes can cause defects in mitochondrial function and activity because mitochondrial dynamics involve multiple processes such as fission and fusion equilibrium, mitochondrial turnover, and motility. However, motility and turnover depend on fusion and fission [114]. Indeed, in neurons, defects in both fusion and fission lead to reduced mitochondrial motility and neuronal dysfunction [114]. In summary, mitochondrial dynamics impairment seems to be involved in several diseases [104]. 

Furthermore, unbalanced mitochondrial dynamics can alter mitophagy by preventing the elimination of damaged mitochondria. Alterations of this type are found in various age-related diseases such as sarcopenia [115], Alzheimer’s disease [116], obesity, and metabolic alterations [117].

Many proteins involved in mitochondrial fission dysregulate with aging. In old mice, the activity of DRP1 reduces, and this is associated with altered morphology of mitochondria in neurons, skeletal muscle, and oocytes [118,119]. Moreover, in the skeletal muscle of aged mice, there is an increased ratio between MFN2/DRP1 and longer intermyofibrillar mitochondria [120]. In human endothelial cells from old subjects, the downregulation of both DRP1 and FIS1 expression is associated with an elongated mitochondrial network [121]. Moreover, with aging, the expression of MFN2 is also downregulated, suggesting a link between reduced fusion and dysfunction in mitochondrial dynamics [115]. 

Several pieces of evidence obtained in many model organisms suggest that changes in the process of mitophagy can affect health and lifespan. It has been reported that the mitophagy rate in 21-month-old mice is not as high as that observed in 3-month-old mice in the dentate gyrus of the hippocampus [122]. Defects in mitophagy have been observed in the satellite cells of the skeletal muscles of aged humans and mice [123] and in the hearts of aged mice [124]. However, the finding of reduced expression of genes encoding proteins involved in mitophagy in the skeletal muscle of physically inactive but not active older women suggests that exercise while preserving muscle mass may protect mitophagy [125]. 

**Figure 2:**
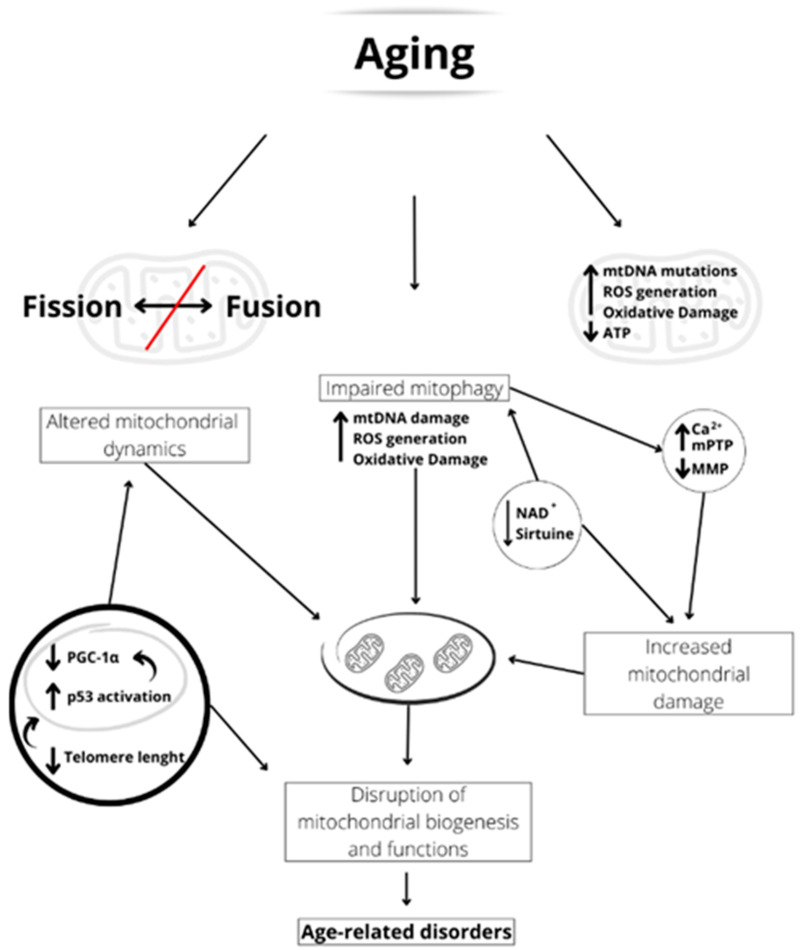
Schematic representation of the aging-linked mitochondrial dysfunctions. Aging can induce unbalanced fusion/fission dynamics, accumulation of mitochondrial damage, and impaired mitophagy. The shortened telomerase length activates p53, which reduces PGC-1α expression and, therefore, mitochondrial biogenesis. Furthermore, reduced NAD+ concentration and sirtuin activity led to impaired mitophagy. This may result in increased mitochondrial [Ca^2+^], mPTP formation, and inner mitochondrial membrane potential (MMP) reduction, leading to increased mitochondrial damage. All these factors induce an alteration of mitochondrial biogenesis and functionality, which are at the basis of age-related disorders.

During aging, both dysfunctional mitochondrial dynamics and inefficient mitophagy can determine the accumulation of damaged and malfunctioning mitochondria in cells, which can impair cellular function (Figure 2). Indeed, it has been reported that the age-related ROS increase in sperm induces mitochondrial damage due to impaired mitophagy [126]. Furthermore, the reduced telomerase length could induce an activation of p53 that suppresses PGC-1α, impairing mitochondrial function and mitochondrial DNA content [127] (Figure 2).

### 4.2. Mitochondrial Biogenesis

The maintenance of efficient mitochondria is necessary for cellular homeostasis and is achieved through the continuous removal of defective mitochondria and the synthesis of new mitochondria (mitochondrial biogenesis) to maintain a mass of good-quality mitochondria to meet cellular energy needs [89]. 

The biogenesis of the mitochondria is a tightly regulated process that involves several transcription factors and the coordination between the mitochondrial and nuclear genomes in a complex and multistep process. The factors involved in the biogenesis process include the nuclear respiratory factors 1 and 2 (NRF1 and NRF2) and transcriptional coactivators, such as members of the peroxisome proliferator-activated receptor γ coactivator-1 family (PGC1α, PGC1β, and PGC1 related coactivator, PRC), of which PGC1α is considered the master regulator of mitochondrial biogenesis, estrogen-related receptors (ERRα, ERRβ, and ERRγ), and mitochondrial transcription factor A (TFAM) [128]. Transcription of the mtDNA starts with the activation, due to either phosphorylation or deacetylation, of PGC1α, followed by stimulation of NRF1, NRF2, and ERRα, with consequent increased expression of TFAM, which acts as the final effector of mtDNA transcription [129].

The translation of genes encoded by mtDNA into proteins requires the participation of translation factors encoded by nuclear DNA: the initiation factors 2 and 3 (mtIF2 and mtIF3), the elongation factors Tu, Ts, and G1 (mtEFTu, mtEFTs, and mtEFG1), the translational release factor1-like (mtRF1L), and the recycling factors (mtRRF1 and mtRRF2). The translational activator of cytochrome c oxidase 1 (TACO1) that binds to the mRNA regulates the levels of mitochondrial proteins [129].

NRF1 and NRF2 directly regulate the expression of nuclear-encoded mitochondrial genes through the production of preprotein [128]. Therefore, the proteins encoded by nDNA derive from preproteins synthesized within the cytosol and containing an amino terminal signal that targets them to the mitochondria. Protein precursors with a cleavable pre-sequence are imported by the pre-sequence translocase (TIM23 complex), while other precursors containing internal targeting signals are imported by the carrier translocase (TIM22 complex) [130]. TIM23 and TIM22 are both activated by the transmembrane electrochemical potential. Many small inner membrane proteins do not resemble canonical TIM23 or TIM22 substrates, and their import mechanism is unknown [130]. 

In summary, both NRF1 and NRF2 are implicated in the expression of mitochondrial respiratory chain proteins, proteins that play the role of promoting the import of preproteins or are involved in the biosynthesis of heme groups, and transcription factors of mtDNA, such as TFAM [128,131]. PGC1α is the master regulator of mitochondrial biogenesis by co-activating the expression of the transcription factors NRF1, NRF2, and ERRα [132].

Several experimental studies suggest that a reduction in mitochondrial biogenesis may be involved in the aging process. It has been found that there is a link between telomere damage and mitochondrial dysfunction [133]. Telomeres are structures possessing repetitive nucleotide sequences and specific binding proteins at the terminal region of the chromosomes of eukaryotes, forming nucleoprotein complexes that assure genomic stability [134,135,136]. In somatic cells, a progressive shortening of telomeres occurs with each division due to the lack of telomerase, an enzyme that adds nucleotides to telomeres. Progressive shortening eventually leads to replicative senescence [137,138], and, in humans, the length of the telomeres in the blood correlates with health and lifespan in 60-year-old or older subjects [139], even if it has been reported that lifespan is mainly related to the percentage of short telomeres rather than the average telomere length [140].

The master regulator of the correlation between telomere length and mitochondrial dysfunction is the nuclear transcription factor protein 53 (p53), which transactivates numerous target genes involved in the induction of cell cycle arrest and/or apoptosis [141,142,143,144], and PGC coactivators of transcription [133]. The shortening of the telomeres is associated with increased p53 and high levels of apoptosis [145]. Indeed, activated p53 promotes the arrest of the cell cycle, or apoptosis, to prevent the propagation of cells with severe DNA damage [146]. Activated p53 can interact with the promoters of *PGC1A* and *PGC1B*, determining the suppression of their expression. The reduction in PGC coactivators determines the reduction in mitochondrial biogenesis and functionality, thus leading to functional decline and aging in tissue stem cells and postmitotic tissues [133]. More importantly, forced expression of PGC1α or deletion of p53 in the context of telomere dysfunction restores mitochondrial respiration [133]. The mitochondrial decline occurring during physiological aging in wild-type mice can be partially reversed by telomerase activation [147], confirming the link between telomere length and mitochondrial efficiency. 

Alterations of mitochondrial biogenesis with age also correlate with a reduction in nicotinamide adenine dinucleotide (NAD^+^) levels [148], which can compromise the activity of sirtuin deacetylases, of which NAD^+^ is a co-substrate (Figure 2) [149]. Seven sirtuin enzymes, from SIRT1 to SIRT7, have been identified in mammals, three of which, SIRT3, SIRT4, and SIRT5, are found in the mitochondrial matrix [149]. The dependence of sirtuins on NAD^+^ makes them dependent on the metabolic state of the cell and sensors of stress [149]. For example, it has been found that SIRT3 increases mitochondrial oxidative metabolism in response to nutrient stress and membrane depolarization [150,151] and that its loss decreases cardiac function and induces neurodegeneration [152,153].

Moreover, SIRT3 activates by deacetylation not only enzymes regulating mitochondrial metabolism, such as isocitrate dehydrogenase or complexes of the mitochondrial respiratory chain, but also enzymes involved in the cellular response to oxidative stress [154].

SIRT3 also contributes to preserving cardiac function in stressful conditions. Indeed, it has been found that the mitochondrial fusion protein OPA1 is post-translationally modified in the heart by hyperacetylation under pathological stress, which reduces its GTPase activity [155]. SIRT3 deacetylating OPA1 elevates OPA1 GTPase activity [155]. The activation of OPA1 by SIRT3 contributes to preserving the mitochondrial network, protecting the cardiomyocytes from doxorubicin-mediated cell death [155].

The involvement of SIRT3 in mitochondrial biogenesis is also suggested by the observation that silencing it in SW620 cancer cells induces decreased mitochondrial biogenesis and mitochondrial dysfunctions, as shown by reduced protein content of PGC1α and TFAM and reduced amounts of the complexes of the mitochondrial chain [156]. These data suggest that SIRT3 regulates mitochondrial homeostasis at several levels, including mitochondrial biogenesis and function. 

Lastly, sirtuins can also modulate autophagy, the cellular-relevant process that allows the lysosomal-mediated elimination of damaged proteins and organelles, including mitochondria, endoplasmic reticulum, and peroxisomes. SIRT1 has been shown to be able to deacetylate p53, resulting in the reduction of apoptosis and the induction of autophagy [157]. Sirtuin-induced activation of autophagy can also reduce cellular senescence, as demonstrated by the effects of SIRT6 overexpression on human bronchial epithelial cells, in which senescence was induced by cigarette smoke extract treatment [158]. Furthermore, the senescence process is increased in both SIRT6 knockdown mice and mice with mutant SIRT6 without histone deacetylase activity [158]. Finally, it has been shown that miR-212-mediated negative regulation of SIRT1-dependent autophagy increases the senescence of cells [159]. In conclusion, it is possible that regulation of the sirtuins that play a role in the biogenesis of mitochondria [160] and the elimination of damaged mitochondria by autophagy [161] can modulate the function of mitochondria and play a protective role in age-associated diseases.

## 5. Mitochondria-Nucleus Communication: Retrograde Signaling and Aging

Damaged mitochondrial elimination by lysosomal autophagy (mitophagy) is a thermodynamically expensive process, so in mitochondria, other quality control processes at the protein or sub-organelle levels are operative, among which the protease system and the “outer mitochondrial membrane-associated degradation” (OMMAD). The first one involves more than twenty proteases [162] localized in both the intermembrane spaces and in the matrix. These play many functions: they regulate the ratios of subunits of mitochondrial complexes that are encoded by nuclear and mitochondrial DNA, eliminate damaged, unfolded, or misfolded proteins, and control protein turnover [163]. OMMAD allows ubiquitinated proteins on the outer mitochondrial membrane to be retrotranslocated to the cytosol for degradation by the proteasome [164]. The removal of damaged mitochondrial components by mitochondrial-derived vesicles (MDVs) or by a form of piecemeal (bit-by-bit) mitophagy is also involved in mitochondrial quality control [164]. MDVs’ removal is a process dependent on Parkin [165] and represents a more cost-effective process with respect to mitophagy [166]. MDVs allow the degradation of not yet depolarized mitochondria, represent a kinetically fast quality control route, and help the clearance of defective mitochondrial proteins and lipids before executing the degradation of the entire organelle by mitophagy [167]. Overall, the involvement of mitophagy, proteases, the ubiquitin–proteasome system, and MDVs is responsible for mitochondrial quality control [164].

**Figure 3:**
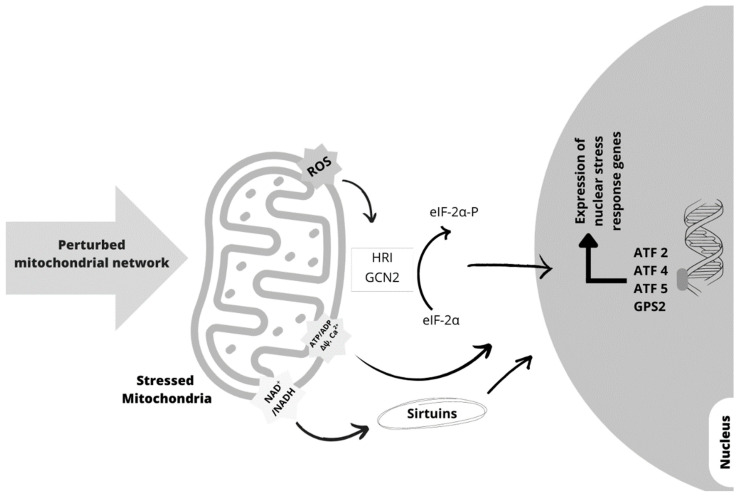
Retrograde signaling pathways. The perturbed mitochondrial network induces an adaptive mechanism. Increased ROS production or mitochondrial dynamic alterations can activate Heme-Regulated Inhibitor (HRI) and General Control Non-derepressible-2 (GCN2), which, in turn, phosphorylate and activate the eukaryotic translation Initiation Factor 2A (eIF-2α). Following its phosphorylation, eIF-2α can induce increased transcription of ATF 2, ATF4, and ATF5, thus activating the nuclear stress-response gene transcription. At the same time, changes in calcium (Ca^2+^), membrane potential (Δψ), ATP/ADP ratio, and NAD^+^/NADH ratio (by a sirtuin-dependent way) can induce the activation of G Protein Pathway Suppressor 2 (GPS2) that can induce the transcription of the nuclear stress-response genes.

A more effective way to control mitochondrial quality is that damaged mitochondria can signal back to the nucleus to orchestrate a nuclear transcriptional response that aims to minimize mitochondrial stress and the resulting damage (retrograde signaling) (Figure 3) [166]. Retrograde signaling comprises evolutionary conserved mechanisms activated by several mitochondrial stresses, including misfolded mitochondrial proteins, increased mitochondrial ROS, altered NAD^+^/NADH and ATP/ADP ratios, calcium levels, and loss of membrane potential (Δψ) [168], that can induce multiple pathways that regulate the communication between mitochondria and nucleus. Each pathway culminates with the activation of many transcription factors, among which, in mammals, the stress-activated transcription factors 4, 5, and 2 (ATF4, ATF5, and ATF2) and G Protein Pathway Suppressor 2 (GPS2), that in the nucleus regulate the expression of nuclear stress response genes translating for mitochondrial chaperones and proteases, ROS detoxification enzymes, and mitochondrial import machinery [168,169] (Figure 3). One of the mitochondrial retrograde signaling pathways in mammals is the unfolded protein response (UPR^mt^), a coordinated response triggered by mtDNA depletion or by protein misfolding in the mitochondrial matrix [169]. The maintenance of the mitochondrial protein folding environment involves many mitochondrial molecular chaperones whose level of expression is dependent on the level of mitochondrial protein homeostasis through the mitochondria-to-nuclear signaling pathway UPR^mt^ [170]. The transcription factor stress-activated transcription factor 1 (ATFS1) regulates UPR^mt^ in *C. elegans*. ATFS1 possesses a sequence necessary for nuclear localization and an amino-terminal sequence that targets it to mitochondria and is essential for the repression of UPR^mt^. In basal conditions, ATFS1 passes into the mitochondria and is degraded. In conditions of mitochondrial stress and dysfunction, the mitochondrial import efficiency is reduced, and the amount of ATFS1 that accumulates in the cytosol and transfers to the nucleus increases [170]. In the nucleus, ATFS1 activates the expression of genes that induce the survival and recovery of the mitochondrial network [169]. In mammalian cells, ATF5 [171] and ATF4 [172] regulate UPR^mt^ in a similar way to the response regulated by ATFS1 in *C. elegans*.

**Figure 4:**
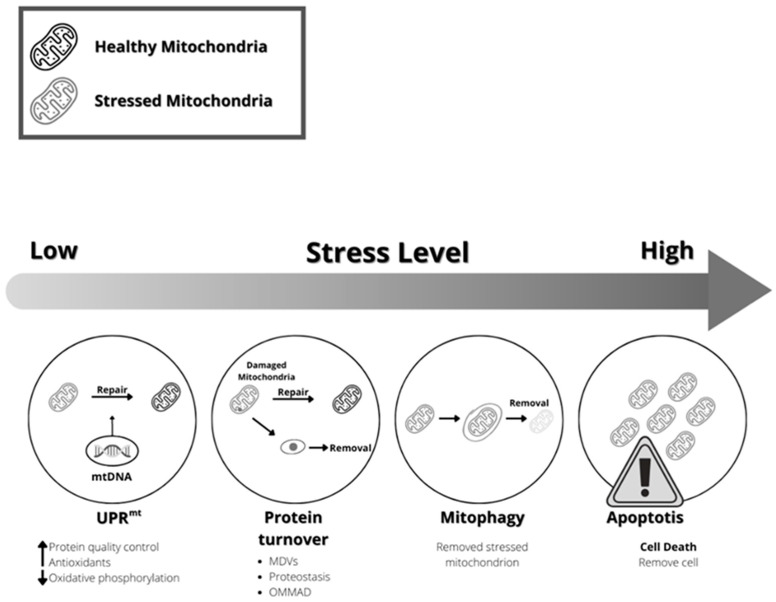
Quality control mechanisms to counteract mitochondrial stress. The mitochondrial response to a stress condition changes with the magnitude of perceived stress. It includes (I) the activation of UPR^mt^ to trigger a transcriptional program to potentially reduce the stress; (II) the removal of damaged proteins by proteases or outer mitochondrial membrane-associated degradation (OMMAD), or of part of damaged portion of mitochondria through mitochondrial-derived vesicles (MDV), to rescue the healthy portion; and (III) mitophagy activation to remove stressed mitochondrion; (IV) induction of cell death to remove the entire damaged cell.

Therefore, cells can perceive the severity of mitochondrial dysfunction and define a response to a danger signal sent by this critical organelle. This response could be the elimination of the entire cell organelle or part of it, the activation of genes coding for proteases, antioxidant enzymes, and proteins involved in protein import into the mitochondria, mitochondrial dynamics, and cell metabolism [169] (Figure 4). 

In age-related diseases and aging, the increased activity of UPR^mt^ allows the protection of mitochondria, preserving their functionality and promoting longevity. A study on *C. elegans* has shown that the expression in the neurons of an aggregation-prone protein that produces aggregates like those seen in Huntington’s disease elicits UPR^mt^ by binding to mitochondria and affects whole-animal physiology [173]. Also, amyloid βeta peptide (Aβ) intraneuronal accumulation evokes UPR^mt^ and mitophagy with a mechanism that is conserved from *C. elegans* to humans and implies the impairment of the import process into the mitochondria, caused by Aβ proteotoxic stress due to an extramitochondrial co-aggregation mechanism [174]. Therefore, enhancing mitochondrial proteostasis to delay amyloid-β proteotoxic diseases can be important to protect against Alzheimer’s disease [175]. 

Finally, many studies show that pharmacological interventions with metformin, resveratrol, rapamycin, or NAD^+^ supplementation that activate retrograde signaling also increase lifespan [176,177,178].

## 6. Aging Mitochondria and Inflammation 

During aging, chronic low-grade inflammation occurs, for which the term “inflamm-aging” was coined. This state is associated with and connected with other factors and diseases characteristic of advancing age [179]. In general, the inflammatory process exerts beneficial actions that allow the neutralization of dangerous or harmful agents, but it can become detrimental in old age [179]. In the inflamm-aging process, the levels of both pro-inflammatory and anti-inflammatory markers increase, and the factors involved are numerous, in addition to the classical cytokines [180]. On the basis of the inflamm-aging process, dysfunctions seem to be involved in the articulated mechanism that removes debris and misfolded and/or misplaced self-molecules from the cell [179].

The damaged molecules, also called damage-associated molecular patterns (DAMPs) [166], bind to a class of specific receptors called pattern recognition receptors (PRRs), which are responsible for unwanted and/or unnecessary inflammatory responses [179]. Mitochondria are sources of DAMPs that potentially stimulate an immune response. Mitochondrial DAMPs include mtDNA, N-formyl peptides generated during the translation of proteins encoded in mitochondria, and the mitochondrial phospholipid cardiolipin [181,182]. Both mtDNA and mitochondrial proteins in the circulation activate an immune response, the first by binding to the Toll-like receptor 9 (TLR9) and the second by binding to the formyl peptide receptor-1 [166].

The amount of mtDNA in the circulation gradually increases after the age of fifty, and inflammatory cytokine levels have been found to correlate with mtDNA levels, being higher in people with higher circulating levels of mtDNA [183]. Moreover, it has been found that the in vitro treatment of monocytes with mtDNA at the highest concentration found in vivo determines an enhancement of the production of the inflammatory cytokine tumor necrosis factor α (TNFα), which demonstrates that mtDNA can induce the production of cytokines [183]. The activation of the inflammatory pathway by mitochondria can also be due to the activation of the "inflammasome", a high-molecular-weight complex present in the cytosol of stimulated immune cells that mediates the activation of inflammatory caspases [184]. Inflammasomes play a crucial role in host defense against pathogens, but their dysregulated activation can favor the development of several diseases, including cancer and autoimmune, metabolic, and neurodegenerative diseases [184]. 

Among the five receptor proteins involved in the activation of inflammasome assembly, leucine-rich repeat (LRR)-containing protein (NLR) 3 (NLRP3) can interact with DAMPs [185], among which mtDNA [186] and the phospholipid cardiolipin [187]. Experimental evidence suggests that the activation of the inflammasome is a more complex event that requires the mitochondria-associated adaptor molecule MAVS for optimal NLRP3 inflammasome activity [188]. Inflammasomes favor the activation of caspase-1, which, in turn, determines the maturation and secretion of pro-inflammatory cytokines IL-1β/IL-18 [185]. Caspase-1 activation increases inflammation by inducing further mitochondrial damage [189]. 

Furthermore, the release of mtDNA can induce the activation of both the inflammasome and mitophagy, and it seems that this last can restrain inflammasome activation through the removal of the damaged mitochondria [190]. 

**Figure 5:**
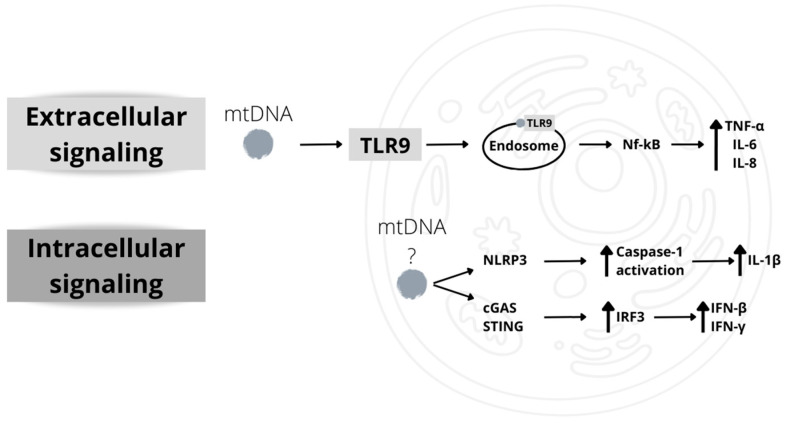
mtDNA and inflammation. Circulating mtDNA binds to the Toll-like receptor 9 (TLR9), which is mainly localized in plasmacytoid dendritic cells, monocytes, B cells, and macrophages and is expressed in the inner face of the endosome membrane. After binding with mtDNA, TLR9 activates an inflammatory response via the nuclear factor kappa-light chain enhancer of activated B cells (NF-kB). In the cytosol of stimulated immune cells, mtDNA can interact with the receptor NLRP3, activating caspase-1, which, in turn, activates the maturation and secretion of pro-inflammatory cytokines IL-1β/IL-18. Alternatively, mtDNA can also bind to the cytosolic sensor of double-stranded DNA (cGAS). Activated cGAS, in turn, activates the adaptor protein STING, leading to the activation of the transcription factor IRF3. IRF3 activates the transcription of genes codifying for IFN-β and IFN-γ.

Alternatively, mtDNA can also be recognized by cyclic GMP-AMP synthase (cGAS), a cytosolic sensor of double-stranded DNA (dsDNA) [166]. Activation of cGAS by dsDNA activates a signaling pathway involving the adaptor protein STING, leading to the activation of the transcription factor IRF3. IRF3 induces the transcription of genes that also participate in viral infection, such as interferon β and γ (IFN-β, IFN-γ) [166]. The involvement of mtDNA in the activation of such a pathway is confirmed by the observation that in embryonic fibroblasts of mice haploinsufficient for the mitochondrial transcription factor TFAM, the cGAS/STING/IRF3 pathway is constitutively activated [191]. This pathway seems to play a significant role in age-related inflammation (Figure 5). 

The mechanism of the release of mtDNA inside and outside the cell is not fully understood, but some data indicate a role for ROS in such a process. In conditions of mitochondrial oxidative stress, the mitochondrial permeability transition pore (mPTP), a component of the mitochondrial inner membrane whose structure is still not fully understood, opens [192]. Through mPTP, a non-selective pore, both ionic and non-ionic substrates can flow, and this has several consequences for the cell, including calcium overload, depolarization of mitochondria, inhibition of ATP synthesis, depletion of pyridine nucleotides, matrix swelling, and cell death if many mitochondria are involved because the swelling causes the rupture of the outer mitochondrial membrane and the release of pro-apoptotic proteins [193,194]. mPTP also constitutes a way for mtDNA to be released. This has been confirmed in studies in which mitochondria from rat livers were treated with Fe^2+^, H_2_O_2_, and calcium ions to induce oxidative stress. In these conditions, the swelling of mitochondria was associated with an increase in the lipid oxidative stress marker thiobarbituric acid and the release of mtDNA [195].

mtDNA is found in several biological fluids, among which are plasma, serum [183,196], cerebrospinal [196], or synovial fluid [197,198]. More than 90% of cell-free mtDNA (cf-mtDNA) is due to intact circulating mitochondria found in healthy subjects in a quantity of about 200,000–3.7 × 10^6^ mitochondria/mL of blood [199,200] that does not have any inflammatory effect. Conversely, free mtDNA can induce inflammation [199,200,201]. Free mtDNA in the extracellular fluid can derive from an active process that is tightly regulated, as happens in neutrophils (NETosis) or other leukocytes, or from dead cell passive release [185]. Neutrophils are the best-characterized sources of actively released mtDNA. They release structures formed of decondensed chromatin, cytosolic, and granule proteins called neutrophil extracellular traps (NETs) in response to bacterial pathogen-associated molecule patterns (PAMPs) [202]. Most of the DNA of the NETs is nuclear, but mtDNA is also present [203]. In some cases, NET production is triggered by ROS, as suggested by the observation that the treatment of neutrophils with the ROS inhibitor diphenyleneiodonium (DPI) inhibits mtDNA release [185] and that ROS-deficient neutrophils do not release mtDNA [185]. These data suggest NET formation and mtDNA release do not depend on cell death but on the presence of ROS [204].

The release of mtDNA in the extracellular medium can be explained by supposing that mtDNA is released into the cytosol and then enclosed in vesicles that fuse with the cell membrane, leading to mtDNA extrusion. Alternatively, a fusion of mitochondrial and cell membranes can occur, leading to the release of mitochondrial content, including mtDNA, in the extracellular space [205]. 

In the aging inflammatory process, the tricarboxylic acid (TCA) cycle metabolite succinate can also play a role. Indeed, the activation of the macrophages by the lipopolysaccharide (LPS) of the Gram-negative bacteria has the effect of inducing a metabolic switch from oxidative phosphorylation to glycolysis, which determines the increase in the cellular level of succinate [206]. A high succinate level stabilizes the hypoxia-inducible factor 1-alpha (HIF-1α) that, in turn, regulates the expression of genes for IL-1β, determining the increase in IL-1β production [206]. Succinate is, therefore, a metabolite in innate immune signaling that leads to enhanced IL-1β production during inflammation [206]. Moreover, succinate can act as a chemokine by interacting with specific succinate receptors coupled to the G protein found on both immune and non-immune cells [207]. Succinate may influence changes in the immune responses that are age-related, which is not surprising considering that in model organisms, including yeasts and worms, levels of TCA metabolites regulate overall life span [208]. 

Succinate accumulation, which could depend on the increase in fumarate accumulation that, in turn, is due to the reduced activity of the enzyme fumarate hydratase (FH) [209], can also induce the succinylation of mitochondrial proteins, which activates the mtDNA release from mitochondria through MDVs. This process has been reported to increase with aging [210]. Another hypothesis is that fumarate accumulation due to FH inhibition can increase mtDNA and mtRNA release by increasing the mitochondrial membrane potential [211]. 

Therefore, the impairment of mitochondrial function, the decline in antioxidant capacity, and increased oxidative stress seem to be deeply involved in aging and age-related diseases [212]. It is possible to hypothesize that if free radicals and other ROS contribute to the accumulation of molecular damage and promote aging and age-related diseases, antioxidants could prevent or delay these processes, prolonging longevity. Therefore, on this basis, many studies have been aimed at estimating the effects on aging and senile diseases of the levels of endogenous antioxidants and/or of the administration of exogenous antioxidants.

Vitamin E is the major liposoluble antioxidant localized in all cellular membranes, including the mitochondrial ones, where it protects the organelles from dysfunction due to increased oxidative damage [35].

## 7. Vitamin E Sources, Adsorption, Antioxidant Properties, and Other Biological Functions

**Figure 6:**
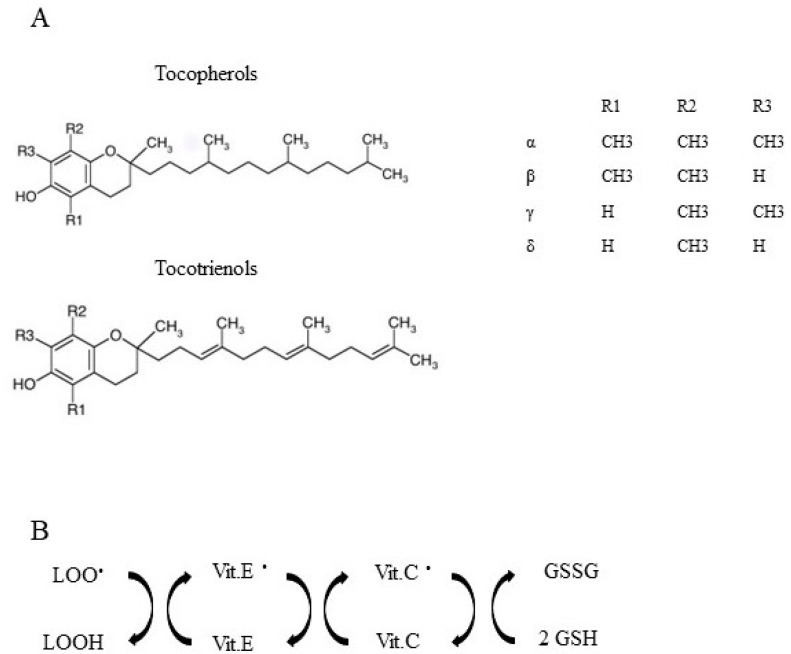
Vitamin E and its biological function. (**A**) different vitamin E isoforms; (**B**) antioxidant activity of vitamin E: conversion of the fat-soluble radicals (LOO^•^) into water-soluble radicals (vitamin C radical, Vit.C^•^). GSH, reduced glutathione; GSSG, oxidized glutathione.

The term vitamin E refers to eight different fat-soluble molecules synthesized in plants, sharing a basic structure consisting of a chromanol ring and an isoprenoid tail (Figure 6), including four tocopherols with saturated tail and four tocotrienols with three double bonds in the tail [213]. Both tocopherols and tocotrienols include four isomers (named α, β, γ, and δ), which differ in the location and number of methyl groups in the chromanol ring (Figure 6). 

Animals acquire vitamin E by consuming vitamin E-rich foods, including oils, nuts, seeds, and wholegrain cereals, and, in human tissues, α-tocopherol is the most represented form of vitamin E [214]. Most dietary vitamin E is free, but supplements usually contain vitamin E esters (α-tocopheryl acetate and α-tocopheryl succinate) because of the greater stability conferred to the reactive hydroxyl group of α-tocopherol [215]. Vitamin E esters are hydrolyzed by gastric lipase or by carboxyl ester hydrolase (also called bile salt-dependent lipase) in the duodenum [215]. Vitamin E absorption is affected by the quantity and quality of the dietary fats [216] and forms micelles in the presence of bile acids, cholesterol, phospholipids, and triacylglycerol to reach the intestinal lumen [217]. Its intestinal absorption is only partially elucidated and occurs by simple diffusion and, at least in part, by cholesterol membrane transporters, the scavenger receptor class B type I (SR-BI), the fatty acids transporter/cluster differentiation 36 molecule (FAT/CD36), Nieman–Pick-C1/like 1 (NPC1L1), and ATP-binding cassettes A1 and G1 (ABCA1 and ABCG1) [218,219].

How vitamin E moves inside enterocytes is a poorly understood aspect. After absorption due to its lipophilicity, it could be transferred to the lipid droplets of the cytosol or the membranes of cell organelles, as suggested by an in vitro experiment in which both α- and γ-tocopherol were found in lysosomes and the endoplasmic reticulum membrane [220] after vitamin E exposure of liver cells. Another possibility is that vitamin E can bind to specific intracellular proteins [221]. In the Golgi apparatus, unesterified vitamin E is packed, together with triacylglycerol, phospholipids, and cholesterol, into chylomicrons, which are released into the lymph [222,223,224]. It appears that vitamin E is also exported via a pathway involving ABCA1 [225] and high-density lipoprotein (HDL) proteins [218,226]. Chylomicrons contain both tocopherols and tocotrienols, both of which are absorbed in the intestine and are transferred from the lymphatic system to the blood via the thoracic duct. Thus, vitamin E reaches peripheral tissues, including bone marrow, adipose tissue, muscle, skin, and the brain [227]. From chylomicrons, vitamin E is transferred to the tissues through a process involving lipoprotein receptors and lipoprotein lipase [227]. In this phase, all the vitamin E isoforms of the chylomicrons can be transferred to the tissues, and therefore, γ-tocopherol localizes in human skin, adipose, and muscle tissues at high concentrations despite its low plasma level [228]. The resulting chylomicron remnants are endocytosed by the liver via a receptor-mediated mechanism involving APOE, which chylomicrons acquire in lymph and blood [229] from high-density lipoproteins [230]. 

In the liver, vitamin E is metabolized. The fate of the different isoforms of vitamin E upon liver uptake is different. Indeed, in the late endosomal compartment, α-tocopherol binds to the α-tocopherol transfer protein (αTTP), localized in the outer leaflet of the endosomal membrane. The interaction between α-tocopherol and αTTP allows its transfer to the plasma membrane, where the complex binds to the phosphatidylinositol 4,5-bisphosphate present therein. This bond causes a conformational change that determines the release of α-tocopherol and its insertion into the membrane [231,232,233]. Subsequently, α-tocopherol binds to ABCA1, and this favors the incorporation of α-tocopherol into lipoproteins that sort it into extrahepatic tissues. Finally, αTTP is recycled via its transfer to the endosomal compartment [231]. α-TTP is highly affine to α-tocopherol (100%), but has a much lower affinity towards β-, γ-, and δ-α-tocopherol (50%, 10–30%, or 1%, respectively) [234]. The binding to α-TTP protects α-tocopherol, which is not metabolized as fast as other forms by CYP4F2 to 13′-hydroxychromanol, 13′-carboxychromanols (13′-COOHs) that are further metabolized via β-oxidation and sulfation to intermediate carboxychromanols, terminal metabolite carboxyethyl-hydroxy chroman (CEHC), sulfated analogues, or conjugated with glucuronic acid and excreted in the feces [227]. 

Vitamin E is essential for humans, and its antioxidant activity protects tissues from lipid peroxidation by transforming fat-soluble radicals into water-soluble radicals (Figure 6) [35] in a reaction chain in which the α-tocopheryl radical is recycled back to α-tocopherol by vitamin C. Therefore, α-tocopherol arrests the chain reactions of lipid peroxidation, neutralizing the radical intermediates that propagate the reaction. In this way, vitamin E protects polyunsaturated fatty acids (PUFA), other components of cell membranes, and low-density lipoprotein (LDL) from oxidation by free radicals [235], and its deficiency appears to be associated with an increased incidence of atherosclerosis and degenerative diseases, as well as a specific disorder called cerebellar ataxia with vitamin E deficiency [236]. It is well known that high levels of plasma cholesterol can determine enhanced oxidation of low-density lipoproteins (LDL), and these can impair endothelial function. In rabbits fed a diet high in cholesterol, vitamin administration prevents atherosclerotic lesions [237,238]. The effects of vitamin E on the progression of atherosclerotic lesions seem to also depend on the dosage used for supplementation and the intrinsic capability to manage lipoproteins. In fact, dietary supplementation of high-dose vitamin E in Watanabe rabbits (2 g of vitamin E/kg of food), which have a rare genetic defect (a deficiency of low-density lipoprotein receptor expression) that predisposes to hypercholesterolemia and hyperlipidaemia [239], reduces both LDL and atherosclerotic lesions [240], while in New Zealand white rabbits fed a cholesterol-enriched diet, vitamin E (10,000 IU of vitamin E/kg of food) does not reduce and on the contrary increases atherosclerotic proliferation of the intima [240]. Moreover, vitamin E limits the generation of ROS and their adverse effects and also reduces inflammatory processes [241]. It has been shown that α-tocopherol reduces the production of pro-inflammatory cytokines and the activation of the nuclear factor kappa-light-chain enhancer of activated B cells (NFkB) [241]. 

Vitamin E has been shown to interfere with the activities of many enzymes, signaling cascades, and gene expression [242]. The enzymes affected by tocopherol include phospholipase A2, cyclooxygenase, and lipoxygenases, both involved in the biosynthesis of lipid mediators or enzymes of signaling pathways, such as protein kinase c α (PKCα) [242]. Other enzymes affected by vitamin E are NADPH oxidase, protein phosphatase 2A, diacylglycerol-kinase, mitogen-activated protein kinase, PH domain leucine-rich repeat protein isoform 1, and phosphatidyl-inositol-3-kinase [242]. The activities of the enzymes can be decreased, and this is the main effect, or increased by vitamin E through mechanisms that are not fully understood [242]. Some of the effects of vitamin E can be attributable to its metabolites. For example, both 13′-COOHs and CEHCs act as inhibitors of cyclooxygenase-1/2 and 5-lipoxygenase, with 13′-COOHs possessing the stronger activity [234]. Moreover, 13′-COOHs were shown to inhibit the growth of cancer cells, regulate the cellular lipid content, and activate the peroxisome proliferator-activated receptor-γ (PPAR γ) and the pregnane X receptor (PRX) [234]. The 13′-COOH metabolites from α-tocopherol and α tocotrienol possess anti-inflammatory and cancer-preventive effects, regulate the microbiota of the gut, and prevent the formation of β-amyloid in mice. Thus, 13′-COOHs are a new class of bioactive compounds with anti-inflammatory and anticancer activities that can potentially modulate lipid and drug metabolism. This suggests that vitamin E metabolites may contribute to the disease-preventive effects exhibited by γ-, δ-tocopherol, and tocotrienols. Conversely, for α-tocopherol, the role of its metabolites could be limited by the fact that most α-tocopherol is associated with αTTP and escapes catabolism by CYP4F2 to a greater extent than other forms of vitamin E [234]. 

Studies conducted on animals and humans demonstrated that γ-, δ-tocopherol, and tocotrienols are metabolized more extensively than α-tocopherol to CEHCs and 13′-COOHs [227]. It has been found that supplementation with pharmacological doses of γT or tocotrienols leads to an increase in CEHCs to >10 μM in the human plasma, with large interpersonal differences in metabolite formation [227]. When αT intake is at the recommended daily assumption (30–50 mg) or below, metabolites formed are minimal. In conditions of relatively high supplementation doses (50 mg or more), metabolites from αT are detected in the plasma and urine [227]. In men diagnosed with localized prostate cancer, supplementation with γT-rich vitamin E mixture significantly elevated the levels of metabolites in prostate tissues [227]. These data show that the metabolites are bioavailable in tissues other than plasma or urine in humans. The metabolites have anti-inflammatory and anti-cancer effects. Furthermore γT, δT, or tocotrienol supplementation attenuates diseases in animal models despite their poorer bioavailability than αT. For instance, γT and δT inhibit colon and breast cancer more effectively than αT in rodents [227].

## 8. Mitochondria and Vitamin E

The main site in which α-tocopherol localizes is within the cell membrane, where it can control lipid oxidation. Because α-tocopherol is present in the membrane at very low levels compared with the high levels of polyunsaturated lipids (PUFA) that are highly susceptible to oxidative attack, the question arises of how the antioxidant can efficiently protect them. To explain such a discrepancy, it has been hypothesized that α-tocopherol localizes into domains (lipid rafts) that are enriched in polyunsaturated phospholipids, thus amplifying the concentration of the vitamin where it is most needed [243]. Moreover, vitamin E is recycled by other antioxidants, among them vitamin C and GSH. 

Interestingly, it has been found that the administered vitamin E accumulates in the flight muscles of a songbird, but only if they regularly exercise [244], suggesting that physical activity can increase the muscle content of the antioxidant. An increase in α-tocopherol content has also been found in the muscle of rats trained to exercise [34]. Therefore, it appears that the muscle content of vitamin E can be increased by physical activity and, plausibly, by the increased activity of mitochondria. 

The localization of α-tocopherol within the membranes of the mitochondria, the major sites of oxidative processes and ROS production, is very important to maintain the oxidative stability of the membrane-bound lipids and prevent damage from reactive oxygen species.

Many studies concerning mitochondrial diseases and dysfunctions have focused their attention on the effects of antioxidant deficiencies and, in particular, vitamin E deficiency [245], while information concerning the effect of the enrichment of mitochondria with antioxidants on cellular health and function is scarce. Studies on the possibility of increasing the concentration of α-tocopherol in the mitochondria were conducted to verify if this increase could improve the quality of the muscle to be used as food. Therefore, most of the data on the effectiveness of a diet enriched with vitamin E is derived from experiments performed on the skeletal muscle of livestock [246]. Indeed, the oxidation of the lipid fraction is mainly responsible for the deterioration of the quality of meat during storage [247,248,249]. In muscle, lipid oxidation seems to start in the highly unsaturated subcellular membranes [249]. Therefore, α tocopherol localization within the phospholipid bilayers of mitochondria and microsomes [250] offers greater protection. α-Tocopherol has been reported to accumulate approximately 6.0- and 8.2-fold more in muscle mitochondria and microsomes, respectively, than in muscle homogenate [251,252].

It seems that a cytosolic binding protein favors the incorporation of α-tocopherol into the mitochondrial [253] and microsomal [254] membranes. Thus, α-tocopherol is most concentrated in cell fractions such as mitochondria and microsomes, and a diet enriched in α-tocopherol increases the content of the vitamin in the subcellular membranes of muscle microsomes and mitochondria of livestock, including pigs, chickens, and cattle [246]. 

The increase in mitochondrial α-tocopherol can also be affected by other diet components, as suggested by the observation that diet supplementation with the combination of copper and all-rac-α-tocopheryl acetate increases the mitochondrial concentration of vitamin E more than all-rac-α-tocopheryl acetate alone [255]. Supplementation with α-tocopherol also increases the vitamin E content in the liver mitochondria [256,257]. The increase in α-tocopherol content protects the mitochondria in conditions of oxidative stress such as hyperthyroidism induced both by thyroid hormone administration (experimental hyperthyroidism) or cold exposure (functional hyperthyroidism) [256,257]. Indeed, in both cases, vitamin E attenuates the hyperthyroidism-linked increase in oxidative damage to lipids and the lowering of the total antioxidant capacities in liver mitochondria [256,257]. 

Furthermore, vitamin E treatment also affects changes in mitochondrial population composition due to oxidative stress. Indeed, the mitochondrial population can be resolved by differential centrifugation into subpopulations that differ in respiratory capacities, ROS production, oxidative damage, and antioxidant capacities, with the heavier population being more functionally efficient but a more significant producer of ROS compared with the lighter fraction.

In the liver of euthyroid rats, the content of the heavier fraction is the highest, while that of the lighter fraction is the lowest [256,257]. In oxidative stress conditions such as hyperthyroidism, the content of the heavier fraction reduces and that of the lighter fraction increases due to the increased removal of the heavier fraction due to the increased oxidative damage [256,257]. The increase in the mitochondrial content of vitamin E due to supplementation prevents the changes in the mitochondrial population distribution, protecting the more efficient fraction from degradation [256,257]. 

Furthermore, in functional hyperthyroidism, α-tocopherol reduces the levels of oxidative damage also in cardiac and skeletal muscle mitochondria [258,259]. The reduced oxidative damage to mitochondria due to vitamin E administration also improves the functional recovery after ischemia and reperfusion of hearts from experimental [258] or functional hyperthyroid rats [260].

## 9. Effects of Vitamin E Supplementation in Aging with a Particular Focus on Mitochondria

The studies concerning the effects of vitamin E supplementation on mitochondria in aging animals are scarce and controversial.

It has been found that diet supplementation with both vitamin C and vitamin E, 10 g/kg and 0.6 g/kg of food, respectively, in rats from 22 to 24 months of age reduces the age-associated oxidation of mitochondrial glutathione [261] in the liver, kidney, and brain. Aging also causes an increase in 8-oxo-7,8-dihydro-2′-deoxyguanosine levels in mtDNA, and oral antioxidant administration also protects against mtDNA damage [261].

Other studies, however, led to different conclusions about the protective effects of vitamin E on older animals. Indeed, the administration of a diet enriched in α tocopheryl acetate (1.65 g/kg) to mice from 21 to 24 months of age [262] enhanced by 3–5-fold the concentration of α-tocopherol in both plasma and tissue homogenates of liver, skeletal muscle, and heart, and by 2–3-fold in their mitochondria. Surprisingly, vitamin E did not reduce the rate of H_2_O_2_ production in mitochondria or the levels of lipid and protein oxidation markers [262]. The same dose of vitamin E failed to reverse the age-associated impairments in cognitive or motor function and the accumulation of oxidative damage in the brains of aged mice [263]. These results suggest that vitamin E supplementation in old mice is not effective in reversing pre-existing age-related impairments of cognitive or motor function and has little effect on protein and lipid oxidative damage in the mouse brain. Furthermore, in aged mice, vitamin E supplementation worsened some brain functions [263]. 

The administration for 10 weeks of a diet containing 1.65 g/kg of α-tocopheryl acetate and 0.72 g/kg of coenzyme Q_10_ [264] to 18-month-old mice ameliorated cognitive and psychomotor impairments due to age, as well as reduced oxidative damage in tissues [264]. In old mice, a diet enriched in Vitamin E and coenzyme Q_10_ improved coordinated running performance. Coenzyme Q10 and vitamin E alone also improved performance, but to a lesser degree [264].

The discrepancies in the results cannot be explained by the different doses of vitamin E administered or by differences in the age at which treatments were started, and currently there is no convincing explanation for the observed differences in the effects of vitamin E administration obtained by different laboratories. Furthermore, long-term feeding of Fischer 344 rats with vitamin E-supplemented diets (500 IU/kg), started when the rats were 6 months of age and continued for 8 months, reduced the age-related deficit in the induction of receptor-mediated signal transduction (striatal muscarinic or cerebellar GABAergic receptor sensitivity) in the central nervous system, which results in behavioral and cognitive deficits [265]. These results suggest that vitamin E can have beneficial effects on cognitive functions if the administration begins in youth and continues in old age and, perhaps, may have some benefit in neurodegenerative diseases [265]. 

Administration of a higher dose of vitamin E (5.0 g α-tocopheryl acetate/kg food) from the age of 28 weeks to mice belonging to a senescence-accelerating strain (CD-1/UCadiz strain inbred at the Department of Experimental Animals of the University of Cadiz), which has a mean life span of 36–57 weeks and a maximum life span of 52–83 weeks, increased the mean lifespan in male mice by 40% and the maximum lifespan by 17%. The same supplementation in female mice increased the mean life span by 14% [266]. It must be underlined that the female mice showed a mean life span longer than males, which agrees with the lower mitochondrial production of oxidants in females than in males and the downregulation of oxidant production by estrogenic hormones [266]. In vitamin E-fed male mice, α-tocopherol content increased 2.5-fold in the brain and 7-fold in the liver. Furthermore, neuromuscular coordination and exploratory activity improved at 52 weeks of age by 9 and 28%, respectively, while at 78 weeks of age they improved by 24 and 45%, respectively [266]. The effects of vitamin E on the behavioral tests were positively correlated with the rates of brain mitochondrial State 3 respiration and activities of Complexes I and IV, whose age-linked decreases were reduced by the vitamin E supplementation [266]. In turn, the rates of respiration, the activities of Complexes I and IV, and the mitochondrial nitric oxide synthetase and MnSOD were negatively correlated with the mitochondrial content of products of the oxidation of lipids and proteins [266]. 

The ability of vitamin E to attenuate the age-induced decline in the respiratory capacity of brain mitochondria has also been confirmed in rats [267]. In this case, the effects of vitamin E supplementation on the whole brain, frontal cortex, and hippocampus were analyzed [267]. In the frontal cortex, there are specialized areas for language and thought, the hippocampus, the median and bilateral formation of the temporal lobe, is specialized for memory and cognitive function and is the area of the brain that is damaged first in Alzheimer’s patients. Both areas undergo metabolic and morphological atrophy in aging rats and humans [268,269]. Dietary supplementation of 9–12-month-old rats with 2.0 or 5.0 g α-tocopherol acetate/kg dose-dependently attenuated the age-related decrease in mitochondrial respiration [267]. Furthermore, in all brain areas, vitamin E also attenuated the age-related decrease in Complex I and IV activities and mitochondrial nitric oxide synthase, with effects being greater with the higher dose [267]. These effects were accompanied by a dose-dependent vitamin E-induced reduction in mitochondrial production of O_2_^−^ and H_2_O_2_ [267]. Interestingly, vitamin E prevented the reduction due to age in mitochondrial mass in the hippocampal CA1 region, as suggested by high-resolution histochemistry of cytochrome oxidase, suggesting that high doses of vitamin E sustain mitochondrial biogenesis in synaptic areas [267].

Vitamin E supplementation also affects other aspects of mitochondrial physiology. In the cerebellar glomeruli of old rats, it has been found that the size of mitochondria increases during aging, and they also appear more elongated with decreased membrane integrity [270]. A similar picture has been found in the hearts of old rats, where the percentage of larger mitochondria has been attributed to increased fusion of the organelles [270,271]. Dietary supplementation with vitamin E (500 IU/kg food) from the age of 540 days to 760 days prevented the age-induced enlargement of the liver mitochondria in mice [272]. Furthermore, by continuing the vitamin E supplementation for up to 870 days, it was observed that aging no longer induced enlargement but only lengthening of the mitochondria. These results suggest that vitamin E provides a protective effect against age-related mitochondrial enlargement [272]. The authors suggest that the age-linked enlargement of mitochondria can depend on the mitochondrial swelling that, in turn, depends on oxidative stress [195], and that vitamin E acting as an antioxidant prevents the process. In Table 1 are resembled the data concerning vitamin E effects on mitochondria in aging.

As stated at the beginning of this paragraph, information regarding the effects of vitamin E supplementation on aging mitochondria is scarce, and data concerning the effects of vitamin E supplementation on mitochondrial dynamics, mitochondrial quality control, and mtDNA-induced inflamm-aging are lacking. Therefore, it would be interesting to study the effects of vitamin E supplementation on the above parameters to get a clear picture of the possibility that it may slow down age-related alterations.

## 10. Conclusions

Even though more than one hundred years have passed since the discovery of vitamin E, the main fat-soluble vitamin in aerobic organisms, many aspects concerning the effects of vitamin E or its metabolites on cellular and mitochondrial physiology are still little studied. Furthermore, vitamin E could play a relevant role in enabling healthy aging in aerobic organisms whose redox homeostasis is continually threatened by ROS production [273].

Finally, emphasizing the importance of vitamin E intake is also crucial, considering the observation that vitamin E intake in the elderly population is often insufficient [274], and deficiency symptoms appear only when it is introduced in very low amounts. So, vitamin E deficiency can be asymptomatic, and it is possible to hypothesize that a low vitamin intake may contribute to increased susceptibility to oxidative stress, which makes elderly people more prone to various types of ailments. Therefore, it seems crucial to control dietary vitamin E intake for healthy aging.

## Figures and Tables

**Table 1:** Effects of vitamin E supplementation on mitochondria in aging.

Ref.	Species Strain	Vitamin E Supplementation	Duration	Tissue	Effects on Mitochondria
[261]	Rat (Wistar)	Vit. E 0.6 g/kg food	From 22 to 24 months	Liver Kidney Brain	↑ GSH ↓ mtDNA damage
[262]	Mouse (C57BL6)	Vit E 1.65 g/kg food	From 21 to 24 months	Liver Skeletal muscle	↔ H_2_O_2_ mit. release ↔ lipid and protein oxidative damage
[266]	Mouse (CD-1UCadiz) (Senescence accelerating strain)	Vit. E 5 g/kg food	From 28 weeks to Death	Brain	↑ mit. State 3 respiration ↑ Complex I and IV activities ↑ MnSOD activity ↑ NOX activity
[267]	Rat (Wistar)	Vit. E 2 g/kg food Vit. E 5 g/kg food	From 9 to 12 months	Total brain Frontal cortex Hippocampus	↑ Complex I and IV activities ↑ NOX activity ↓ H_2_O_2_ mit. Release ↓ O_2_^−^
[272]	Mouse (C57BL/6)	Vit. E 500 UI/kg food	From 540 to 760 days	Liver	↓ mitochondrial enlargement

GSH, reduced glutathione, mit. Mitochondrial, NOX nitric oxide synthase. ↑ increased, ↓ decreased, ↔ unchanged.

## Data Availability

Not applicable.
